# Ductile Zr-Based Bulk Metallic Glasses by Controlling Heterogeneous Microstructure from Phase Competition Strategy

**DOI:** 10.3390/nano9121728

**Published:** 2019-12-03

**Authors:** J. L. Cheng, J. J. Wang, J. X. Rui, Y. L. Yun, W. Zhao, F. Li

**Affiliations:** 1 School of Materials Science and Engineering, Nanjing Institute of Technology, Nanjing 211167, China; wangjingjing142857@163.com (J.J.W.); NJIT_Rjx@163.com (J.X.R.); yunyeling1994@163.com (Y.L.Y.); zw19860610@163.com (W.Z.); 2 Jiangsu Key Laboratory of Advanced Structural Materials and Application Technology, Nanjing 211167, China; 3 State Key Laboratory of Metastable Materials Science and Technology, Yanshan University, Qinhuangdao 066004, China; 4 School of Materials Science and Engineering, Nanjing Tech University, Nanjing 211816, China

**Keywords:** amorphous materials, deformation and fracture, ductility, phase competition

## Abstract

In this paper, we prepare the alloys of Zr_41.2_Ti_13.8_Cu_12.5_Ni_10_Be_22.5_, Zr_44.4_Ti_14.8_Cu_14.3_Ni_11.5_Be_15_, and Zr_38_Ti_12.7_Cu_9.6_Ni_7.7_Be_32_ to show the effects of alloy composition on the inhomogeneity structures and mechanical properties of Zr-based bulk metallic glasses (BMGs). Compared with the best glass former Zr_41.2_Ti_13.8_Cu_12.5_Ni_10_Be_22.5_, some nanoscale inhomogeneity structures can be induced by shifting the compositions towards a primary phase in the alloys of Zr_44.4_Ti_14.8_Cu_14.3_Ni_11.5_Be_15_ and Zr_38_Ti_12.7_Cu_9.6_Ni_7.7_Be_32_. The room temperature compression tests reveal that theBMGs contained nanoscale inhomogeneity structures exhibit superior mechanical properties with the high strength of 1780 MPa and especially a remarkable plastic strain of over 9%. These findings provide a new perspective to enhance the ductility of BMGs by introducing nanoscale inhomogeneity structures based on the phase competition strategy.

## 1. Introduction

Due to the long-range disordering structure characteristic, bulk metallic glasses (BMGs) have some unique mechanical, chemical, and physical properties, which make BMG potentially useful for engineering applications [1,2]. However, most of BMGs usually fail catastrophically at ambient temperature by the highly localized deformation behavior, which severely limits their actual applications. To improve the ductility of BMGs, the BMG composites with the enforcement of intrinsic or extrinsic phases were developed [3,4,5,6,7,8]. These BMG composites exhibited large plasticity, but their yield strengths are decreased significantly. Moreover, some BMGs with nanoscale inhomogeneity structures have been developed, which achieves the combination of high strength and toughness [9,10,11,12]. Therefore, some efforts are devoted to controlling inhomogeneity structures, especially the microalloying technique [13,14,15], composition design from the structural perspective [16] and molecular dynamics simulations [17]. However, it is still hard to predict a priori at what alloy compositions the BMGs would be plastic.

Moreover, it is worth mentioning that a variety of pre-treatments, such as cold rolling [18], high-pressure torsion [19], surface mechanical attrition [20], thermo-mechanical creep [21], thermal cycling [22] and triaxial compression [23], have been used to obtain the inhomogeneity structures and plasticity for the BMGs. These pre-treatments effectively enhance the plasticity of BMGs but also dramatically increase the cost of production. Therefore, it is necessary to propose an effective strategy to design the ductile BMGs.

As known, the origin of inhomogeneity structures in BMGs is correlated with the short-range order and structural fluctuation in the undercooled melting. However, to enhance the glass forming ability (GFA), most of the best glass formers based on the pseudo-binary or ternary eutectics have the highest thermodynamic, dynamic stability and densely stacking structures, which may be detrimental to the formation of nanoscale inhomogeneous structures. Therefore, we proposed that the alloy compositions deviated from the best glass formers could have more amounts of crystal-like short-range order and structural fluctuation, because of the stronger crystallization tendency of the primary phase. In this work, we select the Zr-based alloy system as a model, which has a high GFA and significant scientific and commercial interests [24,25,26]. We will show how to design the BMGs with nanoscale inhomogeneity structures and large plasticity from the phase competition strategy. These findings give a new perspective to enhance the ductility of BMGs.

## 2. Experimental Procedures

According to the authors’ previous studies [27,28], the glass formation of the famous Vit-1 alloy (Zr_41.2_Ti_13.8_Cu_12.5_Ni_10_Be_22.5_) is based on the Zr(Ti)_2_Cu(Ni)–Zr(Ti)Be_2_ pseudo-binary eutectics. To obtain the nanoscaled inhomogeneity structures, we design the two alloy compositions of Zr_44.4_Ti_14.8_Cu_14.3_Ni_11.5_Be_15_ and Zr_38_Ti_12.7_Cu_9.6_Ni_7.7_Be_32_, which are toward the Zr_2_Cu and ZrBe_2_ primary phase respectively. For comparison, the Vit1 alloy was also prepared. Hereafter, the three alloys are designated as Be15, Be22.5, and Be32 respectively. 

Alloy button ingots with the compositions of Zr_41.2_Ti_13.8_Cu_12.5_Ni_10_Be_22.5_, Zr_44.4_Ti_14.8_Cu_14.3_Ni_11.5_Be_15_, and Zr_38_Ti_12.7_Cu_9.6_Ni_7.7_Be_32_ were prepared by arc-melting the mixtures of metal chips with purities higher than 99.9 (wt. %) under a Ti-gettered argon atmosphere. Then the alloy button ingots were remelted and cast into the copper mold with a 7 mm diameter using gravity casting. The microstructures of the samples were investigated by X-ray diffractometry (XRD) and optical microscopy (OM). Cylindrical specimens for uniaxial compression with 3 mm diameter and 6 mm length were machined and conducted on an Instron-8801 testing machine using an engineering strain rate of 5 × 10^−4^ s^−1^. At least three samples for mechanical testing were measured to ensure that the results are reproducible and statistically meaningful. The nanoscaled inhomogeneity structures were investigated by the high-resolution transmission electron microscopy (HRTEM). Differential scanning calorimetry (DSC) was conducted to analyze the thermal properties of the samples under a heating rate of 20 K/min.

## 3. Results and Discussion

**Figure 1:**
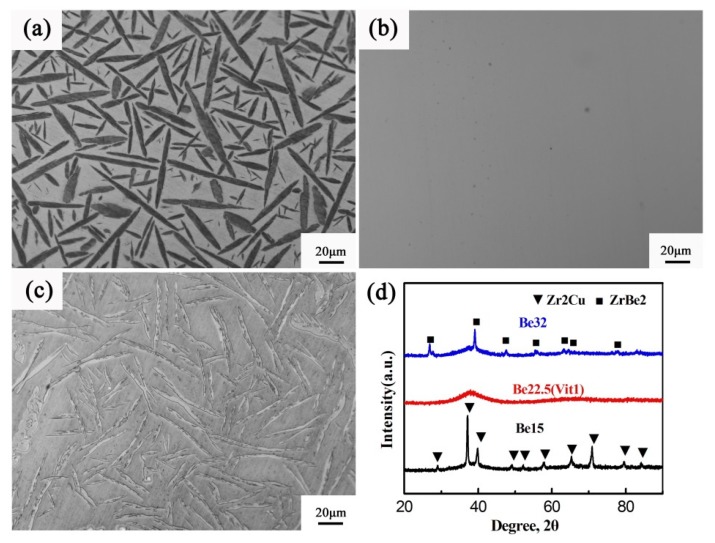
Optical microscopy (OM) micrographs of the master alloys prepared by arc-melting (**a**) Be15, (**b**) Be22.5 (**c**) Be32 and (**d**) their X-ray diffractometry (XRD) patterns.

Figure 1a–c shows the OM micrographs of the three master alloys prepared by arc-melting. As shown, the alloy Be15 exhibits a typical BMG composite, and some needle-like crystals with 45% volume fraction are embedded in the glass matrix. According to the XRD pattern (see Figure 1d), this needle-like phase can be identified as a tetragonal Zr_2_Cu phase. While the alloy Be22.5 shows a featureless amorphous structure, indicating it has the best GFA. For the alloy Be32, it also is a BMG composite with a primary lath-like phase of 29% volume fraction. This lath-like phase can be identified as a hexagonal ZrBe_2_ phase by the XRD pattern. These results indicate that with the increase of Be content, the microstructures of alloys are changed from Zr_2_Cu+BMG to monolithic BMG and then to ZrBe_2_+BMG. Therefore, we propose that in the undercooled melting of alloys Be15 and Be32, there are some origins of inhomogeneity structures, such as crystal-like short-range order structures, which are easy to induce the medium-range order (IMRO) or nanoscale inhomogeneous structures in the alloys of Be15 and Be32, respectively. 

**Figure 2:**
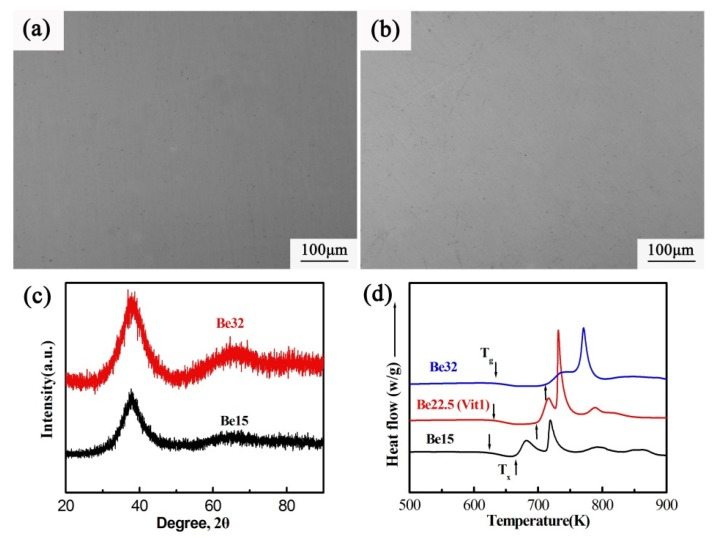
OM graphs of the casting samples of (**a**) Be15, (**b**) Be32 and their correspondence (**c**) DSC curves and (**d**) XRD patterns.

In order to verify the above speculation, the three alloys were cast into a copper mold to obtain rod-like samples with a diameter of 7 mm. Figure 2 shows the OM graphs, XRD patterns and DSC curves of the casting rod-shaped samples. As shown, just like the alloy Be22.5, the alloys Be15 and Be32 exhibit the featureless microstructures (see Figure 2a,b). The amorphous nature of the alloys Be15, Be22.5 and Be32 are also confirmed by the XRD patterns (see Figure 2c), because of them exhibit the broad scattering humps. Moreover, all three alloys show a clear glass transition, further confirming their glass intrinsic nature, as shown in Figure 2d. However, there are obvious differences in the crystallization behavior of the three alloys, which have different onset crystallization temperature *T_x_* and the position of crystallization exothermic peak. It is reasonable to speculate that the different medium-range order structures or nanoscale inhomogeneous structures could lead to this difference in the crystallization behavior.

**Figure 3:**
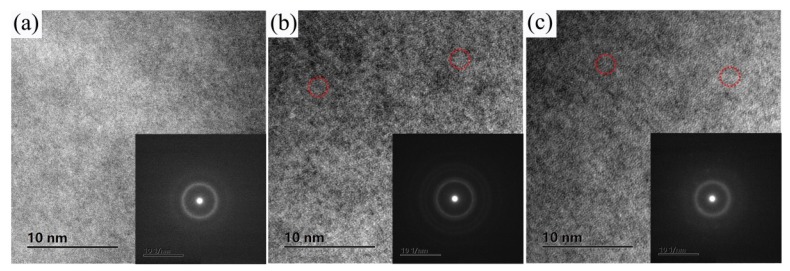
HRTEM images of the casting samples of (**a**) Be22.5, (**b**) Be15 and (**c**) Be32. The insets of (**a**), (**b**) and (**c**) are the corresponding selected area electron diffraction patterns.

TEM was performed to further identify the inhomogeneity structures. As shown in Figure 3, the selected area electron diffraction patterns show that from the region with a diameter of 10 nm consists of a broad halo, characteristic of a fully amorphous phase. Even from the HRTEM image, none of the inhomogeneity structures can be found in the alloy Be22.5, as shown in Figure 3a. However, it seems that there are some nanoscale inhomogeneity structures (denoted by the red circle) exists in the glassy phase for the alloys Be15 and Be32, as shown in Figure 3b,c. Similar phenomenon has been observed in the Zr–Cu–Ni–Al, Zr–Cu–Ni–Al–Pd, Hf–Cu–Ni–Al–Pd, Zr–Pd and Hf–Pd metallic glasses (MGs) [29,30,31], although the IMRO and nanoscale inhomogeneous structures are difficult to identify due to the resolution range of TEMs. It is worth mentioning that Sarac et al. [32] also proposed that with the Ni content increases, the GFA of Fe-based alloy system decreased, but gave rise to nanocrystal formation. Using aberration-corrected high-resolution transmission electron microscopy (HRTEM), they observed the nanoscale inhomogeneity structures on the order of 1–1.5 nm in size. Therefore, we propose that the nanoscale inhomogeneity structures in BMGs can be obtained in the alloys Be15 and Be32, which deviate from the best glass formers and are towards a primary phase.

**Figure 4:**
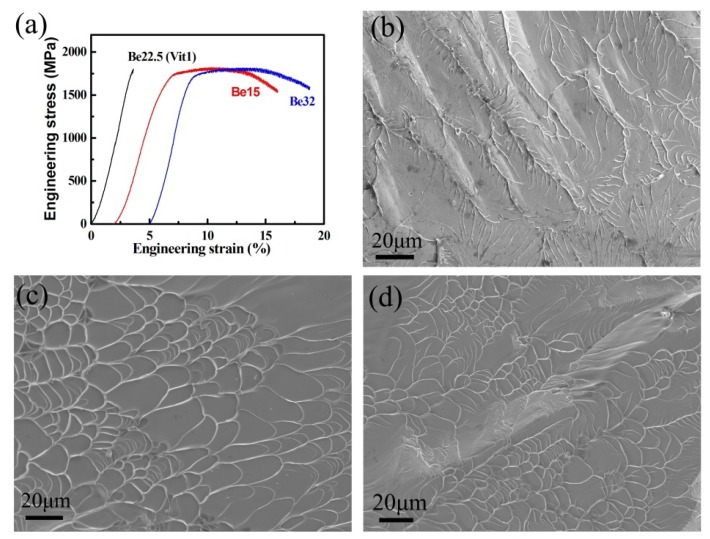
(**a**) Room temperature engineering compressive stress-strain curves of alloys and their fracture surfaces (**b**) Be22.5, (**c**) Be15 and (**d**) Be32.

The room temperature compressive tests were performed to investigate the effect of the nanoscale inhomogeneity structures on the mechanical properties. As shown in Figure 4a, the alloy Be22.5 shows a typical mechanical behavior of BMGs with up to 1790 MPa strength and complete brittle fracture. However, both of the alloys Be15 and Be32 exhibit ~10% plastic strain, while the strengths of them are also up to 1780 MPa, indicating a unique combination of the high strength and large plasticity. In the previous reports [9,32,33], it has been found that the plasticity in BMGs containing fine in-situ precipitated nanocrystals can be greatly improved. In general, the as-casting nanocrystals can stimulate the shear band nucleation and also block the growth of shear bands, and resulting in multiplication of shear bands and macroscopic plastic deformation. Figure 4b–d show the fracture surfaces of the alloys Be22.5, Be15, and Be32, respectively. All three alloys reveal typical vein-like patterns and molten droplets, indicating a local viscous flow during the fracture process. However, it is worthy to notice that the vein patterns are more developed and smaller in the alloys Be15 and Be32, obviously demonstrating its improved plasticity.

## 4. Conclusions

In conclusion, we prepare the BMGs of Be15, Be22.5, and Be32 using copper mold casting. Some nanoscale inhomogeneity structures can be obtained in the BMGs of Be15 and Be32, which are towards the Zr_2_Cu and ZrBe_2_ primary phases, respectively. Compare with the best glass formers Be22.5 without nanoscale inhomogeneity structures, the BMGs of Be15 and Be22.5 exhibit the unique large scale plasticity beside the high strength. These findings give a new perspective to enhance the ductility of BMGs by introducing nanoscale inhomogeneity structures based on the phase competition strategy. Although to some extent this strategy will sacrifice the glass formation ability, it is still a promising method to toughen a BMG for practical engineering applications, especially the excellent glass former system, such as Zr-based BMGs.
